# News and Coming Events

**Published:** 1907-12-14

**Authors:** 


### NEWS AND COMING EVENTS.

The Hospital for Women, Soho.—It has been decided by the Committee of Management, on the recommendation of the Medical Committee, to admit qualified medical women to the practice of the hospital.

---

The Great Northern Central Hospital has received a sum of £10,000 as a donation from Mr. Francis Reckitt.

---

Sir William Bennett, K.C.V.O., has been elected President of the Incorporated Institute of Hygiene in place of the late Sir William Broadbent, Bart.

---

A sub-committee of the Medical Committee of Guy's Hospital has been appointed to consider the whole question of the administration of anæsthetics at this Hospital. Another death under anæsthetic occurred at Guy's Hospital last week.

---

At the National Hospital for the Paralysed and Epileptic, Queen Square, Bloomsbury, W.C., a marble bust of Dr. Hughlings Jackson has recently been unveiled. The bust bears an inscription recording Dr. Jackson's valuable services to the hospital which he has served as physician, and his distinguished contributions to neurology.

---

Royal Society of Medicine (Therapeutical and Pharmacological Section), 20 Hanover Square, W.—Tuesday, December 17, at the Apothecaries' Hall, Blackfriars, E.C., Dr. James Mackenzie, "The Action of Digitalis on the Human Heart." Dr. William Soper, "Reminiscences of an Apprentice Fifty Years Ago."

---

On the recommendation of the House Committee, the Governors of St. George's Hospital have voted a gratuity of 70 guineas a year for three years to the late matron of the hospital. Miss Smedley's services extended over a period of eleven years only, and this precludes the award of a permanent pension to her.

---

In order to utilise the valuable clinical field provided by the Queen Alexandra's Military Hospital, the Army Council has decided to associate that hospital with the Royal Army Medical College, and to obtain the assistance and co-operation of certain acknowledged leaders of the civil medical profession as consultants in medicine and surgery, whose ripe experience and professional skill in another field will be of great benefit to the Army medical service, and will conduce to the efficiency of the hospital as regards both the treatment of the sick and the investigation of the various diseases incidental to military life. The following appointments to the staff have been approved :—To be Consulting Surgeons : A. E. Barker, Esq., F.R.C.S., Professor of Surgery, University College of London; A. A. Bowlby, Esq., C.M.G., F.R.C.S., Surgeon to St. Bartholomew's Hospital; and G. H. Makins, Esq., C.B., F.R.C.S., Surgeon to St. Thomas's Hospital. To be Consulting Physicians : Dr. J. Mitchell Bruce, F.R.C.P., Consulting Physician Charing Cross Hospital; Dr. J. Kingston Fowler, F.R.C.P., Physician to Middlesex Hospital; and Dr. W. Osler, F.R.S., F.R.C.P., Regius Professor of Medicine, University of Oxford.

The provision of fire-extinguishing appliances for hospitals is an absolute necessity in view of the helplessness of the inmates, and the Governors of such institutions may be relied upon to realise their responsibilities in this connection. The North Staffs Joint Hospital Board have just procured a Merryweather manual fire engine for the smallpox hospital at Bagnall. This engine is suitable for use by four men and is equipped with all necessary appliances for working, including an ample supply of delivery hose. In the event of an outbreak of fire at the hospital this engine should prove very valuable.

---

The next meeting of the Medico-Legal Society will be held in the rooms of the Royal Asiatic Society, 22 Albemarle Street, W., on Tuesday evening, December 17, 1907, at 8.15. President, the Hon. Mr. Justice Walton. 1. Narration of Cases and Exhibits of Medico-Legal Interest. 2. "The Radical Cure—Certification of Inebriates," T. Claye Shaw, M.D. Visitors are invited to attend. Any member who is willing to contribute to the proceedings of this session should communicate with the Hon. Secretaries, Messrs. Stanley B. Atkinson, 10 Adelphi Terrace, W.C., and Digby Cotes-Preedy, 2 Elm Court, Temple, E.C.

---

The quarterly court of Governors of the London Hospital was held on December 4, Mr. Sydney Holland presiding. The committee reported that the Queen, President of the hospital, since the last meeting had sent a donation of £666 13s. 4d. to the general funds of the institution. In recommending for election two dental surgeons, the committee had considered it advisable that the department should be opened on every week-day instead of only four times a week. The fire which occurred at the hospital laundry on October 31 did not do much damage, and was speedily extinguished by their own fire brigade. In connection with that matter all the recommendations suggested by Sir Eyre M. Shaw had been carried out. While it was satisfactory to note that the hospital was not in debt, the cost of the upkeep was £100,000 a year, and the assured income from investments and other funds was scarcely £20,000. Without the promissory income there was a balance of £40,000 a year which had to be raised.

